# Lung Superimposed Pressure in Pediatric Acute Respiratory Distress Syndrome: Single-Center Study, 2014–2024

**DOI:** 10.1097/PCC.0000000000004003

**Published:** 2026-07-01

**Authors:** Lorenzo Ball, Matilde Maxenti, Alberto Giardina, Simon Corrado Serafini, Maria Beatrice Damasio, Rocco Grillo, Nicolò Patroniti, Maurizio Cereda, Nadir Yehya, Andrea Moscatelli

**Affiliations:** 1 Department of Surgical Sciences and Integrated Diagnostics (DISC), Università degli Studi di Genova, Genova, Italy.; 2 Anesthesia and Intensive Care, Ospedale Policlinico San Martino, IRCCS Azienda Ospedaliera Metropolitana, Genova, Italy.; 3 Neonatal and Pediatric Intensive Care Unit, Emergency Department, IRCCS Istituto Giannina Gaslini, Genova, Italy.; 4 Department of Intensive Care, Amsterdam UMC, location “AMC,” Amsterdam, The Netherlands.; 5 Radiology Unit, IRCCS Istituto Giannina Gaslini, Genova, Italy.; 6 Department of Anesthesia, Critical Care and Pain Medicine, Massachusetts General Hospital, Boston, MA.; 7 Department of Anesthesia, Critical Care, and Pain Medicine, Harvard Medical School, Harvard University, Boston, MA.; 8 Division of Pediatric Critical Care Medicine, Children’s Hospital of Philadelphia, Philadelphia, PA.; 9 Department of Anesthesia and Critical Care Medicine, Perelman School of Medicine, University of Pennsylvania, Philadelphia, PA.

**Keywords:** acute respiratory distress syndrome, mechanical ventilation, pediatric acute respiratory distress syndrome, pediatric, superimposed pressure

## Abstract

**OBJECTIVES:**

Superimposed pressure (SP) is the hydrostatic pressure compressing dependent lung regions and is a major determinant of dorsal collapse in adult patients with acute respiratory distress syndrome (ARDS). CT studies clarify its role in determining the need for positive end-expiratory pressure (PEEP) in ARDS. We therefore aimed to describe SP in pediatric ARDS (pARDS) and assess its associations with anthropometric, clinical, and quantitative CT parameters.

**DESIGN:**

Retrospective cohort study.

**SETTING:**

Urban academic tertiary care children’s hospital in Italy.

**PATIENTS:**

All children with pARDS undergoing noncontrast chest CT between 2014 and 2024 compared with age-matched control patients with normal lung findings undergoing chest CT.

**MEASUREMENTS AND MAIN RESULTS:**

We included 12 pARDS patients and 24 controls. Quantitative CT analysis was used to measure lung aeration and the SP in 10 ventral-to-dorsal regions. The median (interquartile range [IQR]) SP increased from ventral to dorsal in both groups but was higher in pARDS patients: (dorsal SP, 5.9 cm H_2_O [IQR, 4.4–7.5 cm H_2_O] vs. 3.0 cm H_2_O [IQR, 2.4–4.0 cm H_2_O]; *p* < 0.001). Compared with controls, we found that pARDS patients had higher lung weight, lower gas/tissue ratio, and less normally aerated tissue. In pARDS patients, SP correlated with chest circumference (ρ = 0.60; *p* = 0.043), lung weight (ρ = 0.76; *p* = 0.006) and inversely with normally aerated tissue (ρ = –0.72; *p* = 0.011). We failed to identify a relationship between SP and oxygenation indices.

**INTERVENTIONS:**

None.

**CONCLUSIONS:**

SP in pARDS is lower and more variable compared with descriptions in adult patients with ARDS and is both affected by disease severity and chest size. We failed to identify an association between oxygenation indices and SP, which raises concerns about titrating PEEP based on oxygenation targets alone. Further studies are needed to confirm and integrate these in pARDS.

**RESEARCH IN CONTEXT**

- In adults with acute respiratory distress syndrome (ARDS), CT studies showed that superimposed pressure (SP) drives dorsal collapse and contributes to positive end-expiratory pressure (PEEP) requirements.
- Comparable evidence of SP and dorsal lung collapse in pediatric ARDS (pARDS) is lacking.
- We therefore studied the hydrostatic forces in children with and without pARDS, examining how chest geometry from CT and loss of aeration contributed to SP magnitude and variability.

**AT THE BEDSIDE**

- Our chest CT findings show that SP in pARDS patients is lower compared with previous reports in adults, more variable, and associated with chest size and lung weight.
- However, we failed to identify an association between oxygenation indices and SP, which suggests that oxygenation-based PEEP titration may have pitfalls.
- Further studies are needed to evaluate optimal PEEP selection in pARDS; perhaps individualized by patient size, disease extent, and oxygenation impairment assessment.

Although acute respiratory distress syndrome (ARDS) can occur at any age (1), pediatric ARDS (pARDS) is now a distinct entity (2, 3), requiring specific considerations due to heterogeneity in biology, respiratory support, and treatment (4). Furthermore, epidemiology from PICUs in 27 countries, 2016–2017 (5), Asia 2017–2023 (6), and Spain 2019–2021 (7, 8) shows that mortality remains high. In this context, protective invasive mechanical ventilation is a cornerstone to management, typically involving low tidal volume, low plateau pressure, and the use of positive end-expiratory pressure (PEEP) (6, 9–12). In pARDS, the strategy for PEEP titration during mechanical ventilation (2, 13, 14) is largely extrapolated from evidence in adult patients with ARDS. However, a key difference here between adults and children is lung size and its impact on aeration loss, which differs from that observed in CT studies in adult ARDS patients (15, 16).

A concept that is enabled by a noncontrast chest CT scan is an entity called the superimposed pressure (SP), which is the hydrostatic pressure exerted by the weight of overlying lung tissue in the dorsal, dependent regions of the lung (17). The SP is determined by lung weight and size, and it is the main reason for dorsal lung collapse in ARDS (18). Hence, in patients with ARDS, the application of PEEP has the possibility of counteracting alveolar collapse induced by SP and restoring aeration (19); it also follows that support with PEEP level below the SP is associated with persistent dorsal lung collapse (20). Since there is a paucity of chest CT studies in cases of pARDS, we aimed to review our use of this investigation and describe SP in the pediatric population. We hypothesized that, in pARDS patients, the magnitude of SP is associated with chest size and severity of lung disease. We also sought to evaluate the associations between SP, patient size, and the degree of oxygenation impairment.

## METHODS

We performed a retrospective cohort study (institutional review board [IRB] study protocol “Lung superimposed pressure in pediatric acute respiratory distress syndrome” number 0034946/24; approved October 31, 2024), with the need for written informed consent waived, because of the retrospective nature of the study. Throughout our research work and handling of patient data, we followed the ethical standards of our IRB, and our research complied with the Helsinki Declaration of 1975.

Eligible subjects were identified between January 2014 and December 2024 from Istituto di Ricovero e Cura a Carattere Scientifico (IRCCS, Scientific Institute for Research, Hospitalization and Healthcare) Giannina Gaslini (Genoa, Italy). During this time, part of our standard practice was to obtain CT scans in select cases of pARDS. For this study, patients with pARDS and controls for comparison were identified using local databases held by the PICU and the hospital’s radiology picture archiving and communication system (PACS). Our study inclusion criteria were as follows. In potential cases in the pARDS group, these were admission to the PICU, 0–16 years, noncontrast chest CT without relevant motion artifacts, and available mechanical ventilation and oxygenation data. The diagnosis of pARDS was retrospectively confirmed by two pediatric intensivists (A.M. and A.G.) using the Pediatric Acute Lung Injury Consensus Conference group (PALICC-2) 2023 updated criteria (2). The control group was identified by finding pediatric patients who had undergone chest CT as part of extrathoracic cancer staging, with normal chest findings.

Clinical parameters collected from the local databases and medical records included patient age, comorbidities, anthropometry, ventilation, and oxygenation data. When necessary, clinical documentation was retrieved from the hospital’s remote archive and manually reviewed. Ventilation and oxygenation data were collected from the records on the same day of CT acquisition at a time closest to the timing of the CT scan. We included pARDS patients with data that allowed us to compute four oxygenation parameters: 1) the oxygenation index (OI); 2) the ratio of the Pao_2_ to Fio_2_ (Pao_2_/Fio_2_ ratio); 3) the ratio of the pulse oximetry oxygen-hemoglobin saturation to Fio_2_ (Spo_2_/Fio_2_ ratio); and 4) the Pao_2_/Fio_2_ ratio corrected for PEEP (PF/PEEP ratio), which is computed as [Pao_2_×10]/[Fio_2_×PEEP] (21).

### Image Analysis

Chest CT scans were retrieved from the PACS and stored in raw Digital Imaging and Communications in Medicine format, without any additional postprocessing. The CT scans were acquired on a multidetector scanner (Sensation 64, Siemens Healthineers, Erlangen, Germany) using standard chest protocols; images were reconstructed with B60f, B31f, or B30f convolution kernels, with median reconstruction diameter 282 mm (IQR, 231–293 mm), median pitch 1.3 (IQR, 1.2–1.4), median tube voltage 100 kVp (IQR, 100–100 kVp), median tube current–time product 114 mAs (IQR, 88–203 mAs), slice thickness 0.625 or 1.0 mm, median in-plane pixel spacing 0.55 mm (IQR, 0.45–0.57 mm), and median field of view 282 mm (IQR, 231–293 mm).

Chest circumference was measured on the CT scan with a polygonal line on an axial slice at mid-chest level to approximate the clinical measurement of thoracic circumference obtained at the bedside using a measuring tape. Lung antero-posterior diameter was measured at its maximum extension.

Lung parenchyma segmentation was performed using ITK-SNAP (Version 3.8.0,University of Pennsylvania, Philadelphia, PA, www.itksnap.org) with a semi-automatic algorithm followed by manual refinement with exclusion of airways and vessels with a diameter greater than 5 mm. This approach is standard practice in quantitative CT studies of ARDS and shows good interoperator agreement (22). Quantitative CT analysis was conducted in MATLAB (R2024a, The MathWorks, Natick, MA) with custom-written scripts. We computed the following whole-lung parameters based on established methods (23, 24): total lung volume, total lung weight, gas volume, tissue volume, and gas/tissue ratio; and the percentage of hyperaerated (i.e., Hounsfield Units [HU] < –900), normally aerated (i.e., –900 ≤ HU < –500), poorly aerated (i.e., –500 ≤ HU < –100), or nonaerated compartments (i.e., HU ≥ –100) expressed as a percentage of the total lung weight. The gas/tissue ratio was defined as ${\;}^{G}/{\;}_{T}=\frac{V_{gas}}{V_{tissue}}=\frac{HU}{-1000-HU}$, where HU is the lung mean attenuation, *V*_gas_ is the lung gas volume, and *V*_tissue_ the lung tissue volume (18). The **Supplemental Digital Content (SDC) 1** (https://links.lww.com/PCC/C753) contains MATLAB pseudocode to allow reproduction of all key computations performed in this study.

### SP and Regional Lung Analyses

The SP was estimated according to approaches described in a previous study (18). We assume the lung parenchyma is a fluid-like medium transmitting hydrostatic pressure, so the pressure at a given vertical level within the lung follows Stevin’s law of hydrostatics and is determined by the weight of the parenchyma lying above that point, that is, its average density multiplied by the distance from the ventral lung surface. Computation was restricted to a basal 9-mm CT slab placed above the diaphragm dome, as in the original definition of SP (18), which aimed at identifying the highest SP in ARDS (25). This CT slab was sectioned into 10 equally spaced ventral-dorsal regions of interest (ROIs) numbered 1 (ventral) to 10 (dorsal). For each ROI, quantitative CT parameters were computed as for the whole-lung analysis, and the hydrostatic pressure was computed as $P_{ROI}=density_{ROI}\times h_{ROI}$, where density is expressed in grams per milliliter computed from ROI attenuation (HU) as $\;density_{ROI}=\;\left(1-\frac{HU_{ROI}}{-1000}\right)$ and *h*_ROI_ is the ROI height in centimeters, namely the distance from the ventral lung surface. This computation provides the hydrostatic pressure (*P*) expressed as cm H_2_O. The SP at a given ROI level was defined as cumulative hydrostatic pressure of that ROI and all overlying ROIs. The overall lung SP, that is, the most dorsal value, was used for correlation analyses. **Figure 1** illustrates the ROIs’ definition and the methods used to compute the SP.

**Figure 1.**
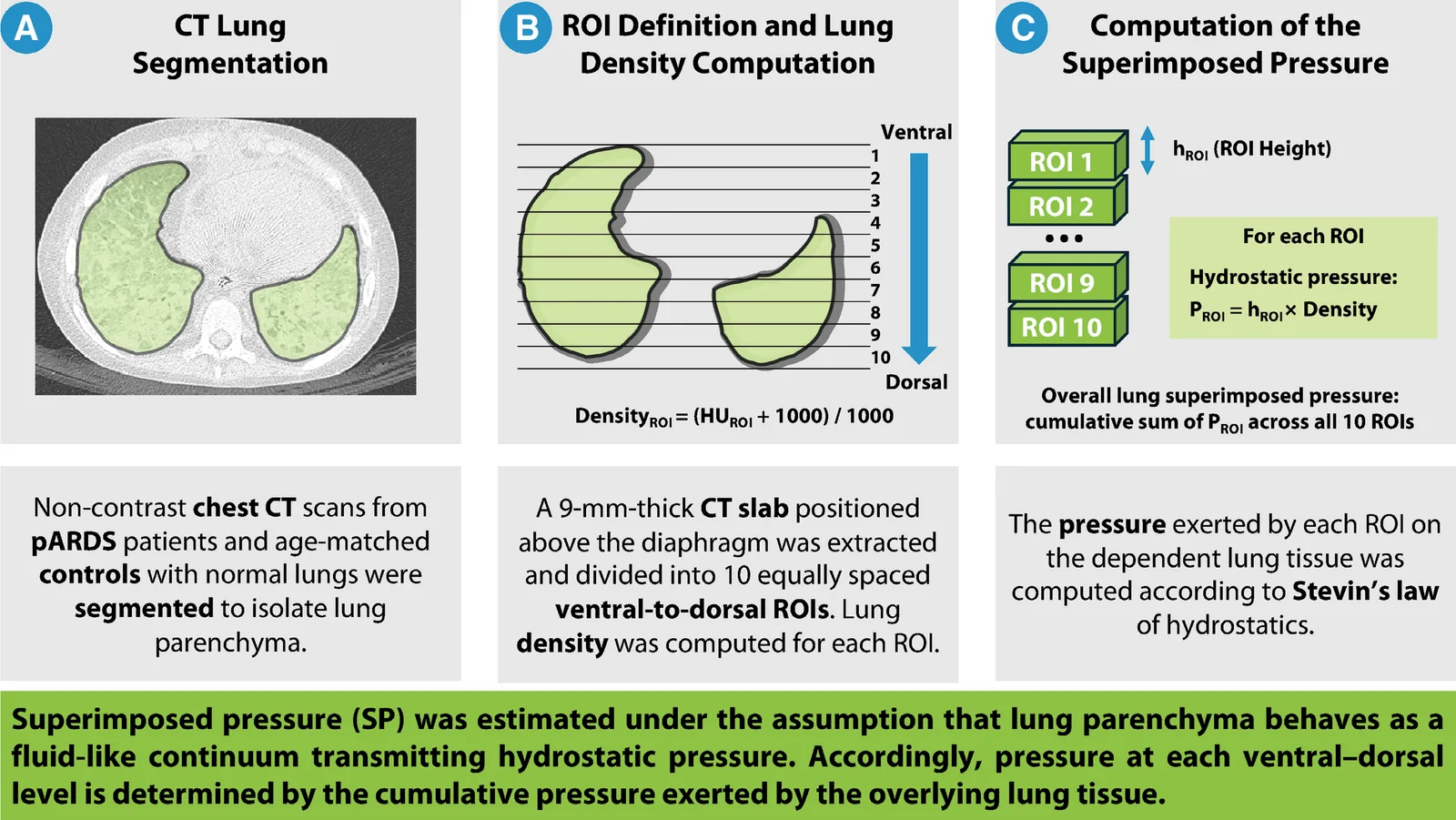
Workflow for the computation of superimposed pressure (SP) from quantitative chest CT analysis. **A**, Lung parenchyma segmentation on a basal noncontrast chest CT slice. **B**, Illustrates extraction of a 9-mm-thick CT slab positioned above the diaphragm and its segmentation into 10 equally spaced ventral-to-dorsal regions of interest (ROIs), with lung density in each ROI computed from CT attenuation. **C**, Depicts estimation of regional hydrostatic pressure computed as *P*_ROI_ = *h*_ROI_ × density_ROI_, where *h*_ROI_ is the vertical distance of each ROI from the ventral lung surface and density_ROI_ is lung density derived from Hounsfield Units (HU). Overall lung SP is computed as the sum of *P*_ROI_ across all ROIs from ventral to dorsal regions. pARDS = pediatric acute respiratory distress syndrome.

### Study Endpoints and Statistical Analyses

The primary endpoint of the study was to describe the distribution and values of SP in pARDS vs. controls. Secondary endpoints included correlations with anthropometric, oxygenation, and quantitative CT parameters.

An a priori sample size computation was not feasible because of the retrospective design, the low prevalence of pARDS, and the exceptional use of noncontrast CT scans in our practice. However, our sample size of 12 patients and 24 controls gave us 80% power to detect correlations of at least |ρ| = 0.72 at an alpha level of 0.05. Matching was performed by age at the time of the CT scan, using the nearest-neighbor matching with a 6-month caliper and a 1:2 case-to-control ratio. Balance after matching was assessed by comparing age distributions between groups. Data are reported as median (IQR), plots were represented as mean ± se of the mean, unless otherwise specified. Differences between groups were assessed with the Mann-Whitney *U* test with Holm-Šídák correction for multiple comparisons, and correlations with Spearman ρ. Effect size was estimated using the Hodges–Lehmann estimator with a 95% CI. To explore the joint contribution of loss of aeration and chest size on the SP, in a sensitivity analysis we used a linear regression model to fit the SP as a function of the chest circumference and percentage of normally aerated lung. This approach was aimed at capturing the effects on the SP of both patient size and extension of lung disease and was carried out after checking residual diagnostics and variance inflation factors. Model coefficients were used to estimate the increase in chest circumference and the percent loss of normally aerated lung tissue associated with a +1 cm H_2_O increase in SP by inverting the corresponding regression coefficients. Data were analyzed using R Version 4.0.3 (R Foundation for Statistical Computing, Vienna, Austria; www.r-project.org), using “*ggplot2*” and “*pwr*” libraries for graphs and power analysis, respectively. A *p* value of less than 0.05 was considered statistically significant.

## RESULTS

Over the 11 years (2014–2024), out of 397 chest CT scans performed in PICU patients, we identified 12 patients with pARDS who we matched with 24 patients with normal lung findings (see **SDC2**, **Fig. S1**, https://links.lww.com/PCC/C754). Children with pARDS and their matched controls had a median of 2 years (IQR, 1–9 yr) and 2 years (IQR, 1–10 yr), respectively (*p* = 0.76). In the pARDS group, median body weight was 14 kg (IQR, 11–25 kg), and the median interval between PICU admission and CT scan was 10 days (IQR, 0–15 d). Consistent with the age-matching strategy, median chest circumference was similar in pARDS vs. controls: 56 cm (IQR, 51–65 cm) vs. 52 cm (IQR, 49–77 cm); *p* = 0.53. Diagnoses in the pARDS cases included sepsis (*n* = 5), pneumonia (*n* = 5), COVID-19 (*n* = 1), and major surgery (*n* = 1). Based on the OI, pARDS severity was classified as mild (*n* = 3), moderate (*n* = 6), and severe (*n* = 3).

### Ventilation, Oxygenation, and CT Data

At the time of the CT scan, pARDS patients were invasively ventilated with a median respiratory rate of 26 min^–1^ (IQR, 24–29 min^–1^), median inspiratory pressure of 22 cm H_2_O (IQR, 20–28 cm H_2_O), median PEEP of 8 cm H_2_O (IQR, 6–10cm H_2_O), and median Fio_2_ of 0.5 (IQR, 0.4–0.7). The acid-base and gas exchange values were as follows: median pH 7.38 (IQR, 7.33–7.46), median Pao_2_ 61 mm Hg (IQR, 51–69 mm Hg), and median Paco_2_, 48 mm Hg (IQR, 41–58 mm Hg). The hemodynamic parameters were median heart rate 118 min^–1^ (IQR, 98–124 min^–1^) and median mean arterial pressure 67 mm Hg (IQR, 53–77). The oxygenation indices were median OI, 10 (IQR, 7–14); median Pao_2_/Fio_2_, 108 mm Hg (IQR, 86–150 mm Hg); median Spo_2_/Fio_2_, 192 (IQR, 138–238), and median PF/PEEP 127 (IQR, 95–273). A structured summary of clinical and physiologic variables at the time of CT acquisition is provided in SDC 2 (**Table S1**, https://links.lww.com/PCC/C754). As detailed in SDC 2 (**Table S2**, https://links.lww.com/PCC/C754), lung size was similar between groups. However, pARDS cases had higher lung weight, less normally aerated tissue, higher nonaerated mass, and lower gas-to-tissue ratio.

### SP and Regional Analyses

As shown in **Figure 2**, the estimated SP increased progressively from the ventral to the dorsal regions in both pARDS patients and controls, as expected due to the hydrostatic behavior of the lung parenchyma. The estimated SP was higher in the pARDS group compared with controls in the four most dorsal ROIs. In the most dorsal region, median estimated SP was 5.9 cm H_2_O (IQR, 4.4–7.5 cm H_2_O) and 3.0 cm H_2_O (IQR, 2.4–4.0 cm H_2_O) in the pARDS and control groups, respectively (median difference, 2.6 cm H_2_O; 95% CI, 1.5–4.1 cm H_2_O; *p* < 0.001).

**Figure 2.**
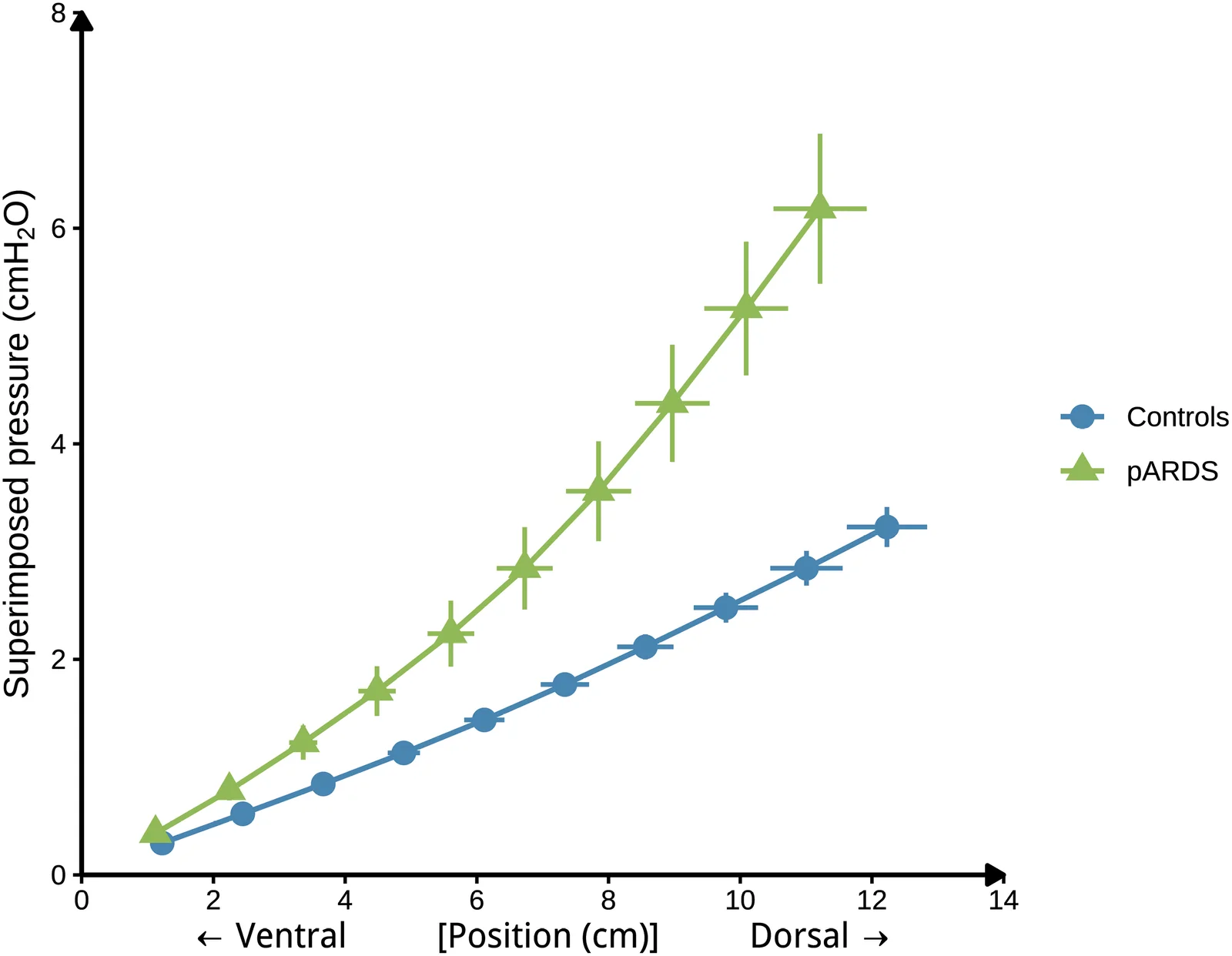
Superimposed pressure (SP) plotted against position along the ventral-dorsal axis for pediatric acute respiratory distress syndrome (pARDS) patients and controls. Data are shown as mean ± se of the mean for each region of interest. “*” indicates SP is significantly higher in pARDS vs. control at *p* < 0.05.

From the ventral to the dorsal region, the gas/tissue ratio decreased in both pARDS patients and controls, with a steeper decrease in pARDS, as illustrated in **Figure 3**. The proportion of nonaerated and poorly aerated tissue increased in pARDS from the ventral to the dorsal ROIs, while remaining substantially stable in controls (SDC 2, **Fig. S2**, https://links.lww.com/PCC/C754).

**Figure 3.**
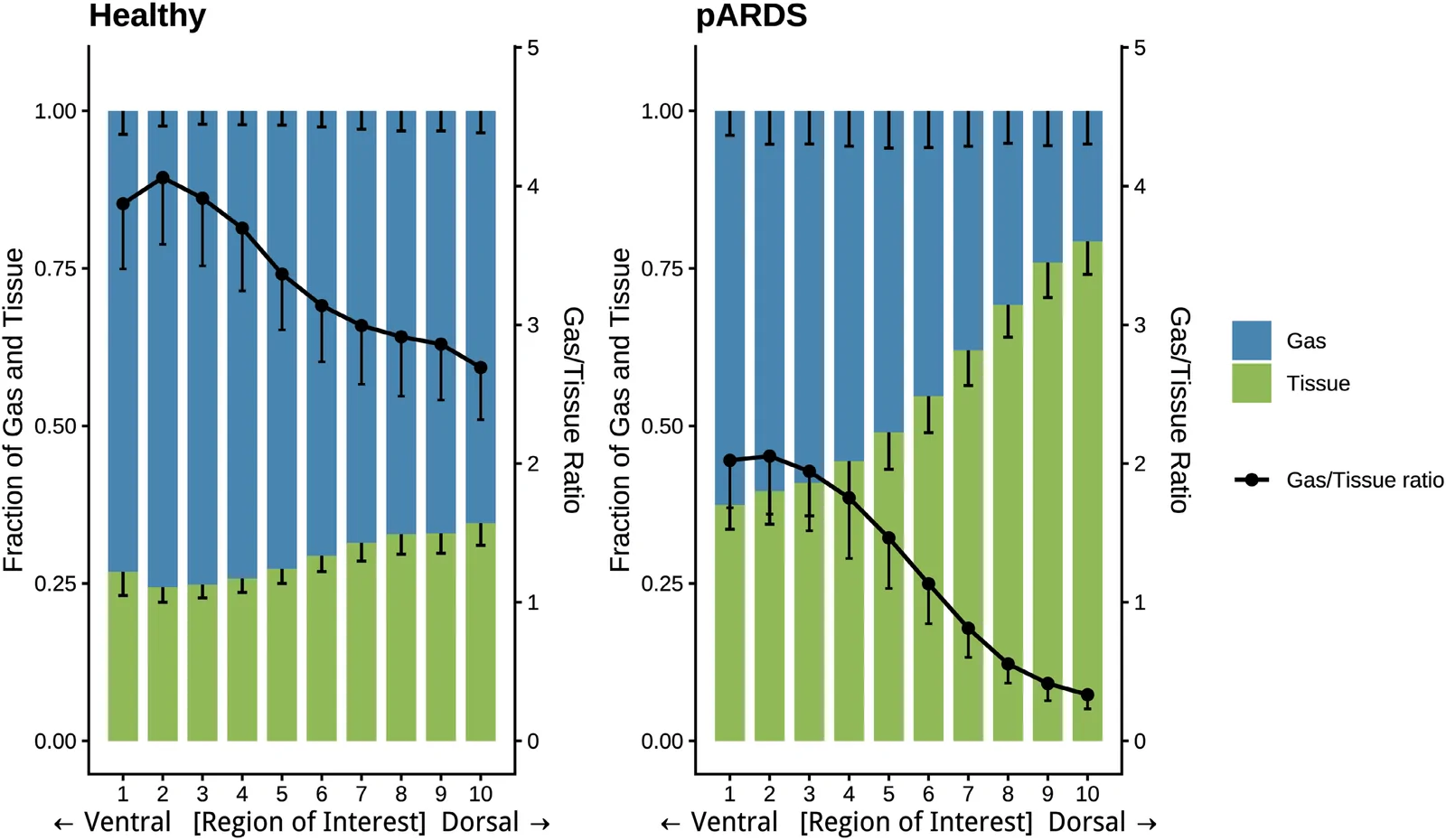
Fraction of gas and tissue volume and the corresponding gas/tissue ratio for each ventral-dorsal region of interest in pediatric acute respiratory distress syndrome (pARDS) patients and controls. Data are mean ± se of the mean.

### Correlations with Clinical and CT Parameters

Correlation analyses were conducted only in pARDS patients, as anthropometric and oxygenation data were not available for controls. Chest circumference was correlated with SP (ρ = 0.60; *p* = 0.043), while we failed to observe a correlation with the following: age, weight, the antero-posterior lung diameter (**Fig. 4**), and the oxygenation parameters (i.e., OI, Pao_2_/Fio_2_ ratio, Spo_2_/Fio_2_, and PF/PEEP ratio; see **Fig. 5**). Among quantitative CT parameters (SDC 2, **Fig. S3**, https://links.lww.com/PCC/C754), SP was correlated with the total lung weight (ρ = 0.76; *p* = 0.006) and inversely with normally aerated tissue (ρ = –0.72; *p* = 0.011).

**Figure 4.**
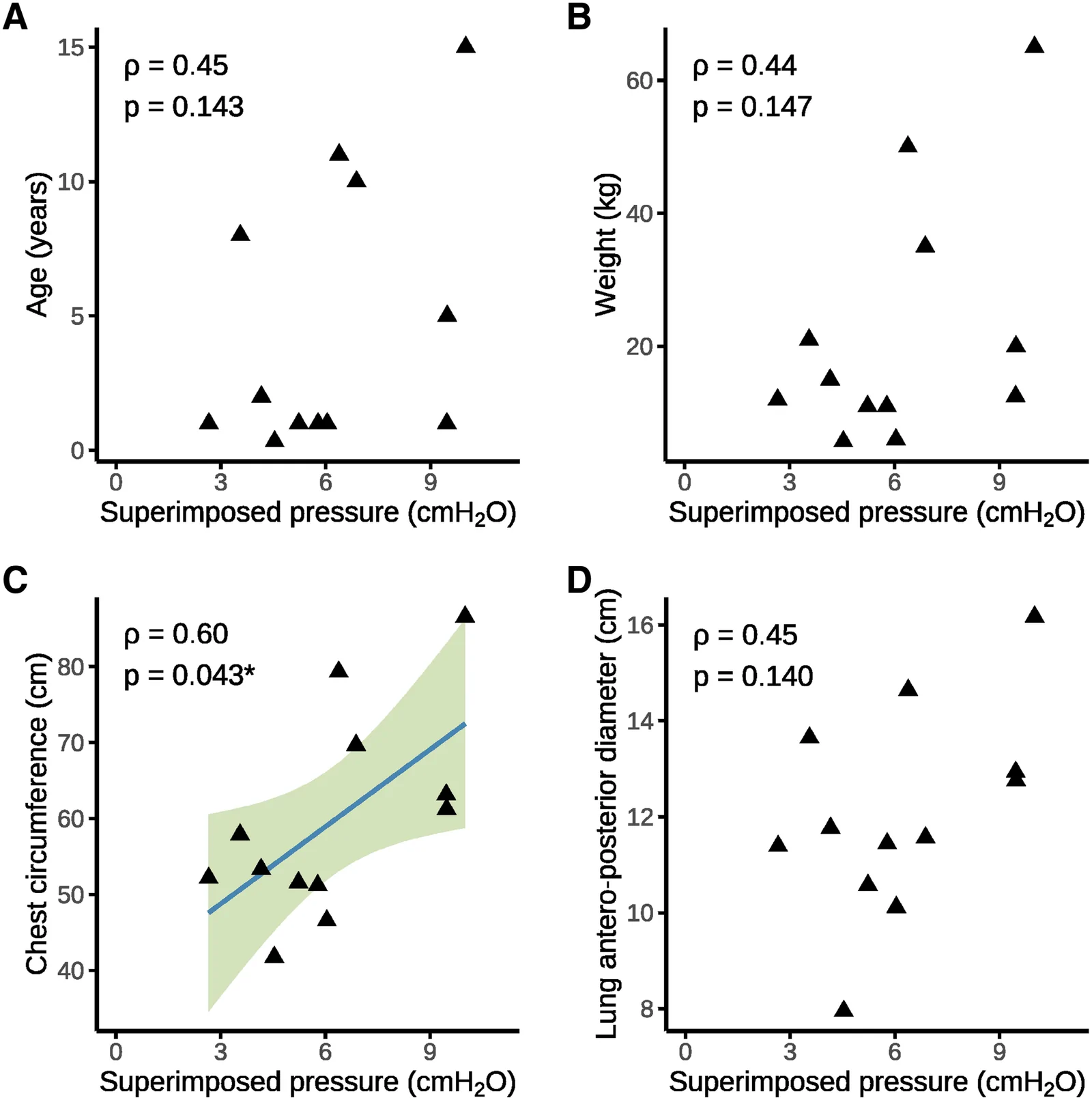
*Scatterplots* showing the relationship between superimposed pressure in the most dorsal region of interest and anthropometric measures in pediatric acute respiratory distress syndrome patients. *Line* represents linear regression with 95% confidence bands.

**Figure 5.**
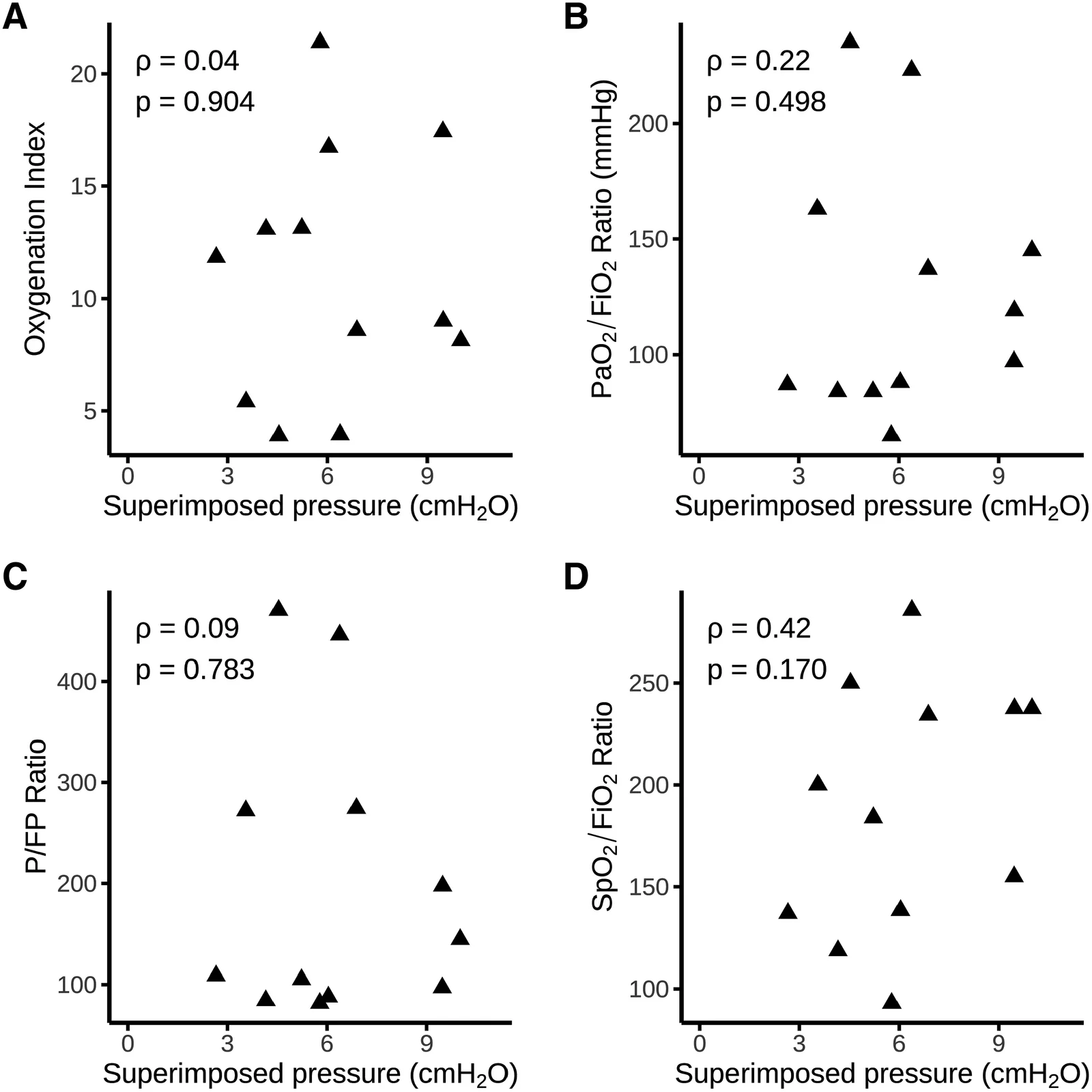
*Scatterplots* of superimposed pressure in the most dorsal region of interest vs. oxygenation parameters (Pao_2_/Fio_2_, oxygenation index, pulse oximetry oxygen saturation/Fio_2_ ratio, and ratio of Pao_2_/Fio_2_ divided by positive end-expiratory pressure). We failed to identify any significant correlations.

Last, in the exploratory linear regression modeling of SP as a function of circumference and loss of normally aerated lung tissue, the combination of these two factors explained 81% of the SP variability in this cohort (adjusted *R*^2^ = 0.81). Larger chest circumference was related to higher SP (β = 0.09; *p* = 0.005), whereas a greater proportion of normally aerated tissue was associated with lower SP (β = –0.14; *p* < 0.001). Therefore, an increase of 11 cm in chest circumference or a 7% loss of normally aerated tissue each corresponded to a +1 cm H_2_O increase in SP.

## DISCUSSION

In this single-center cohort study of lung SP in cases of pARDS, we have made five key observations using CT scans in 12 cases (and 24 age-matched controls) over the 11 years, 2014–2024. First, the estimated SP increased from ventral to dorsal regions in both pARDS and control patients. Second, SP values were higher in pARDS patients than in controls. Third, the overall SP values in pARDS patients were heterogeneous and overall lower than in adult patients with ARDS. Fourth, we failed to identify a correlation between oxygenation indices and SP. Last, SP was correlated with extension of lung injury and children’s chest size. Taken together, these findings extend the adult evidence about management of severe respiratory failure with consideration of SP to the pediatric population. However, there are important differences related to body size and disease heterogeneity.

In our pediatric cohort from 2014 to 2024 the median estimated SP in 12 pARDS patients was 5.9 cm H_2_O with three-quarters of the patients having an SP less than 7.5 cm H_2_O. In adults, one of the first reports on SP published in 1994 (with 17 ARDS patients and 12 controls) estimated that the magnitude of SP was around 10 cm H_2_O (18), whereas a study of 51 adults with ARDS published in 2014 showed that the magnitude of SP is relatively homogeneous across patients and largely dictated by lung density (25). In children, growth and therefore age-related variability in chest size and thoracic geometry introduces a factor not identified in the studies in adults with ARDS. Hence, in children, this variable should be considered in addition to lung disease severity as an influence on the magnitude of SP. Chest circumference also appeared to be better correlated with SP than the anteroposterior diameter, whereas we failed to identify associations with age or weight in this small cohort. This finding may reflect that chest circumference captures overall thoracic geometry, including rib cage size and chest wall mechanics, which can modulate how regional lung weight is transmitted as compressive forces within the thorax. This idea that chest morphology may not reflect SP is consistent with data from a 2016 to 2022 cohort of obese adult patients with ARDS (26), as well as an experimental study of PEEP in lung-injured, 3–4-month-old pigs carried out in 2024 (27).

In our study, we failed to find a correlation between the oxygenation indices and the CT-derived, model-estimates of SP, possibly also because of the small sample size. However, if these observations are confirmed in larger physiologic studies, then there are important clinical implications for individualizing PEEP in pARDS patients, possibly suggesting caution in relying on oxygenation indices alone. Indices using Spo_2_ and Pao_2_ are, by definition, required in the contemporary diagnosis and stratification of disease severity in pARDS (2); even so, they may not be reflective of the CT-derived estimate of SP. This inability to find a correlation may have been influenced by our lack of a standardized approach to mechanical ventilation during chest CT scan acquisition. This deficiency could be remedied by a prospective study with planned ventilation settings, including PEEP. In adult patients with ARDS, CT-derived PEEP requirements and SP (18) are relatively constant across patients, regardless of the degree of lung recruitment (25), and patients with lower Pao_2_/Fio_2_ ratios receive higher PEEP levels for optimizing gas exchange (28). In contrast, in patients with pARDS, the assumption that lung density is the major determinant of SP may be incorrect since the child’s chest size may also be an important determinant of SP and, indirectly, of PEEP requirement. The 2014 PALICC-1 report advised grading pARDS according to disease severity (i.e., by degree of impaired oxygenation) and included a strong recommendation on the use of high PEEP levels in severe pARDS cases (29). The 2023 PALICC-2 update (2) issued a conditional recommendation on PEEP optimization above the levels identified according to the ARDS Network’s lower PEEP/higher FiO_2_ table. These recommendations will need to be integrated with usual physiologic reasoning (30, 31) and include patient considerations of chest size and underlying disease to determine optimal PEEP requirements. Here, emerging bedside techniques, such as electrical impedance tomography, may help translate these CT-derived concepts into clinical practice (32, 33).

Our study has some limitations. First, the sample size was too small to ensure detection of weaker associations; this problem was a consequence of noncontrast chest CT being rarely indicated in children with pARDS, allowing us to analyze only 12 pARDS patients over a period of 11 years (i.e., ~1 per year). Second, the estimation of SP using chest CT measurements and calculations relies on the assumption that the lung parenchyma behaves like a fluid medium. Therefore, the derived SP values should be interpreted with caution. Third, our retrospective cohort study required retrospective adjudication of pARDS diagnosis, and we did not have direct, real-time measurement of transpulmonary pressure (14, 34). Last, quantitative CT analysis has two intrinsic main limitations: 1) it is affected by manual segmentation, and 2) the lung aeration metric relies on the assumption that the HU is a linear reflection of the average amount of water and lung tissue within a voxel (i.e., sub mm^3^ scale). Nonetheless, quantitative chest CT measures do reflect macroscopic lung characteristics (35).

In conclusion, our single-center, retrospective cohort study of 12 pARDS patients managed over a period of 11 years (2014–2024) shows that SP shares the same pathophysiological role as described in adult patients with ARDS. However, in comparison with adult patients, our data show lower estimates of SP with high between-patient variation. Importantly, SP in children with pARDS is not associated with lung disease only but also with chest size, a factor largely absent from descriptions of adult patients with ARDS. Furthermore, we failed to find associations between oxygenation indices and the mechanical forces related to SP, which may suggest limitations in relying solely on oxygenation for PEEP titration. Therefore, larger, prospective physiologic studies and bedside monitoring tools are needed to integrate these concepts into clinical decision-making.

## Supplementary Material
