# Supplementary material for: Lung Superimposed Pressure in Pediatric Acute Respiratory Distress Syndrome: Single-Center Study, 2014–2024
Source: Pediatr Crit Care Med. 2026 Jul 1;27(10):1244–53. doi: 10.1097/PCC.0000000000004003 (PMC13641260; doi:10.1097/PCC.0000000000004003)
Supplement: Supplementary file 1 [file pcc-27-1244-s001.pdf]

```
==============================================================================

MATLAB-style pseudocode for superimposed pressure and quantitative CT
Lorenzo Ball et al. 2026
CT arrays are [slices x rows x cols]

REQUIRED VARIABLES (assumed defined upstream):
  ImageHU            : full CT data (HU), [Nslices x Nrows x Ncols]
  LungMask           : lung parenchyma segmentation mask, same size
  voxelSizeMm        : [dr dc] in-plane pixel spacing (mm)
  sliceThicknessMm   : slice thickness from DICOM (mm)
  slabThicknessMm    : desired basal slab thickness (9 mm)
  DiaphragmSliceN    : index of slice at diaphragm dome
  Nrois              : number of ventral–dorsal ROIs (10)

Ventral–dorsal axis:
  - corresponds to COLUMNS (dimension 3)
```

Contents

- ==
- ------------------------ Basal slab selection --------------------------
- ------------------------ Define voxel geometry -------------------------
- ------------------------ Ventral–dorsal ROI definition -----------------
- ------------------------ Density from HU -------------------------------
- ------------------------ Hydrostatic pressure & SP ---------------------
- ------------------------ Gas and tissue volumes ------------------------
- ------------------------ Lung weight and aeration compartments ---------

==

------------------------ Basal slab selection --------------------------

```
Identification of the thick slab above the diaphragm
```

```
% Number of slices required to cover the desired slab thickness
nSlabSlices = ceil(slabThicknessMm / sliceThicknessMm);

% Define slab immediately above the diaphragm
kEnd   = DiaphragmSliceN;
kStart = DiaphragmSliceN - nSlabSlices + 1;

% Restrict analysis to basal slab
ImageHU  = ImageHU(kStart:kEnd,:,:);
LungMask = LungMask(kStart:kEnd,:,:);
```

------------------------ Define voxel geometry -------------------------

```
dr_cm = voxelSizeMm(1)/10;        % row spacing (mm)
dc_cm = voxelSizeMm(2)/10;        % column spacing (mm) → ventral–dorsal
dz_cm = sliceThicknessMm/10;      % slice thickness (mm)

voxelVolume_mL = dr_cm * dc_cm * dz_cm;    % 1 cm^3 = 1 mL
```

------------------------ Ventral–dorsal ROI definition -----------------

Identify columns containing lung tissue, then splits into equally spaced ventral-to-dorsal ROIs

```
LinCols = squeeze(any(any(LungMask,1),2));   % [Ncols x 1]
```

```
ventralCol = find(LinCols,1,'first');
dorsalCol  = find(LinCols,1,'last');

nColsLung = dorsalCol - ventralCol + 1;

% Contiguous ROI edges
edges = round(linspace(0, nColsLung, Nrois+1));

ROI_mask      = cell(Nrois,1);
ROI_height_cm = zeros(Nrois,1);

for i = 1:Nrois

    c1 = ventralCol + edges(i);
    c2 = ventralCol + edges(i+1) - 1;

    if i == Nrois
        c2 = dorsalCol;
    end

    ROI_mask{i} = false(size(LungMask));
    ROI_mask{i}(:,:,c1:c2) = LungMask(:,:,c1:c2);

    % Height from ventral surface to ROI midpoint
    colMid = (c1 + c2)/2;
    ROI_height_cm(i) = (colMid - ventralCol) * dc_cm;
end
```

----------------------- Density from HU ------------------------------

HU → density (g/mL): density = 1 + HU/1000 Compute for all ROIs

```
% Initialize mean HU and ROI density vectors
ROI_mean_HU     = zeros(Nrois,1);
ROI_density_gml = zeros(Nrois,1);

% Compute for all ROIs
for i = 1:Nrois
    hu = ImageHU(ROI_mask{i});
    ROI_mean_HU(i)     = mean(hu);
    ROI_density_gml(i) = 1 + ROI_mean_HU(i)/1000;
end
```

----------------------- Hydrostatic pressure & SP ---------------------

Hydrostatic increment: dP = density × height (cmH2O)

```
% Compute hydrostatic pressure in each ROI
ROI_dP_cmH2O = ROI_density_gml .* ROI_height_cm;

% Cumulative superimposed pressure summing pressure of each ROI
ROI_SP_cmH2O = zeros(Nrois,1);
for i = 1:Nrois
    ROI_SP_cmH2O(i) = sum(ROI_dP_cmH2O(1:i));
end
```

----------------------- Gas and tissue volumes -----------------------

Tissue fraction ≈ density; gas fraction = 1 − density Volumes obtained by voxel-wise summation

```
% Initialize output vectors
ROI_tissueVolume_mL = zeros(Nrois,1);
ROI_gasVolume_mL    = zeros(Nrois,1);
ROI_gasTissueRatio  = zeros(Nrois,1);
```

```
% Compute tissue volume, gas volume and gas-to-tissue ratio in each ROI
for i = 1:Nrois
    hu  = ImageHU(ROI_mask{i});
    rho = 1 + hu/1000;

    tissueFrac = rho;
    gasFrac    = 1 - rho;

    ROI_tissueVolume_mL(i) = sum(tissueFrac) * voxelVolume_mL;
    ROI_gasVolume_mL(i)    = sum(gasFrac)    * voxelVolume_mL;
    ROI_gasTissueRatio(i)  = ROI_gasVolume_mL(i) / ROI_tissueVolume_mL(i);
end
```

----------------------- Lung weight and aeration compartments -----------

Voxel mass: mass_g = density × voxelVolume_mL

Aeration compartments (HU): Hyper-aerated: HU < -900 Normally aerated: -900 ≤ HU < -500 Poorly aerated: -500 ≤ HU < -100 Non-aerated: HU ≥ -100

```
% Initialize output vectors
ROI_totalMass_g  = zeros(Nrois,1);
ROI_pctHyper     = zeros(Nrois,1);
ROI_pctNormal    = zeros(Nrois,1);
ROI_pctPoor      = zeros(Nrois,1);
ROI_pctNon       = zeros(Nrois,1);

% Compute aeration compartments as % of the total lung mass
for i = 1:Nrois
    hu  = ImageHU(ROI_mask{i});
    rho = 1 + hu/1000;
    m   = rho * voxelVolume_mL;

    ROI_totalMass_g(i) = sum(m);

    ROI_pctHyper(i)  = 100 * sum(m(hu <  -900)) / ROI_totalMass_g(i);
    ROI_pctNormal(i) = 100 * sum(m(hu >= -900 & hu < -500)) / ROI_totalMass_g(i);
    ROI_pctPoor(i)   = 100 * sum(m(hu >= -500 & hu < -100)) / ROI_totalMass_g(i);
    ROI_pctNon(i)    = 100 * sum(m(hu >= -100)) / ROI_totalMass_g(i);
end
```

Published with MATLAB® R2024a
