# Supplementary material for: Lung Superimposed Pressure in Pediatric Acute Respiratory Distress Syndrome: Single-Center Study, 2014–2024
Source: Pediatr Crit Care Med. 2026 Jul 1;27(10):1244–53. doi: 10.1097/PCC.0000000000004003 (PMC13641260; doi:10.1097/PCC.0000000000004003)
Supplement: Supplementary file 2 [file pcc-27-1244-s002.pdf]

SUPPLEMENTAL DIGITAL CONTENT 2

Figure S1

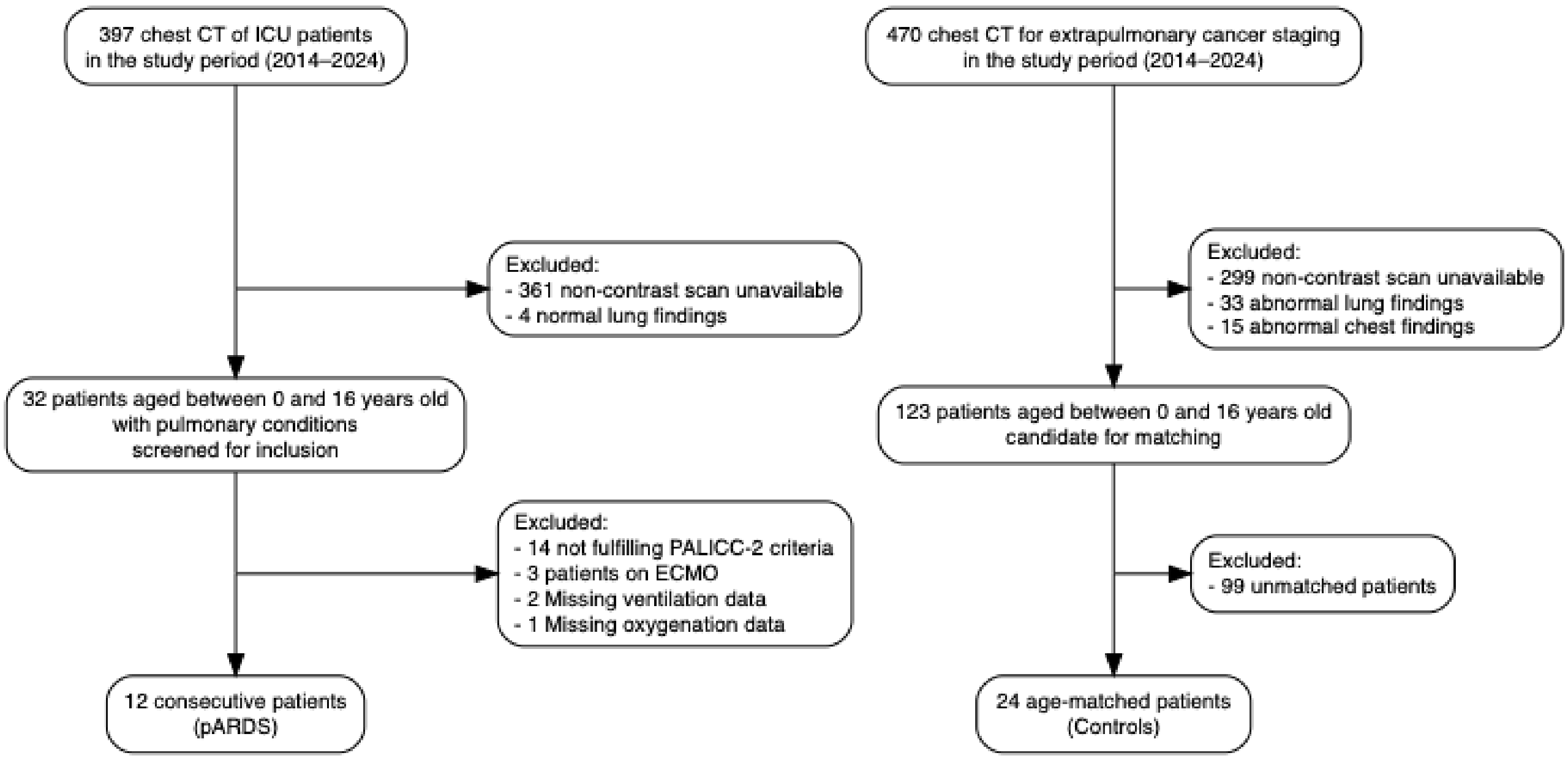

Figure S1: Patient inclusion flow chart. Where: pARDS is Pediatric Acute Respiratory Distress Syndrome; CT is Computed Tomography; ICU is Intensive Care Unit; and PALICC-2 is the Second Pediatric Acute Lung Injury Consensus Conference.

Figure S2

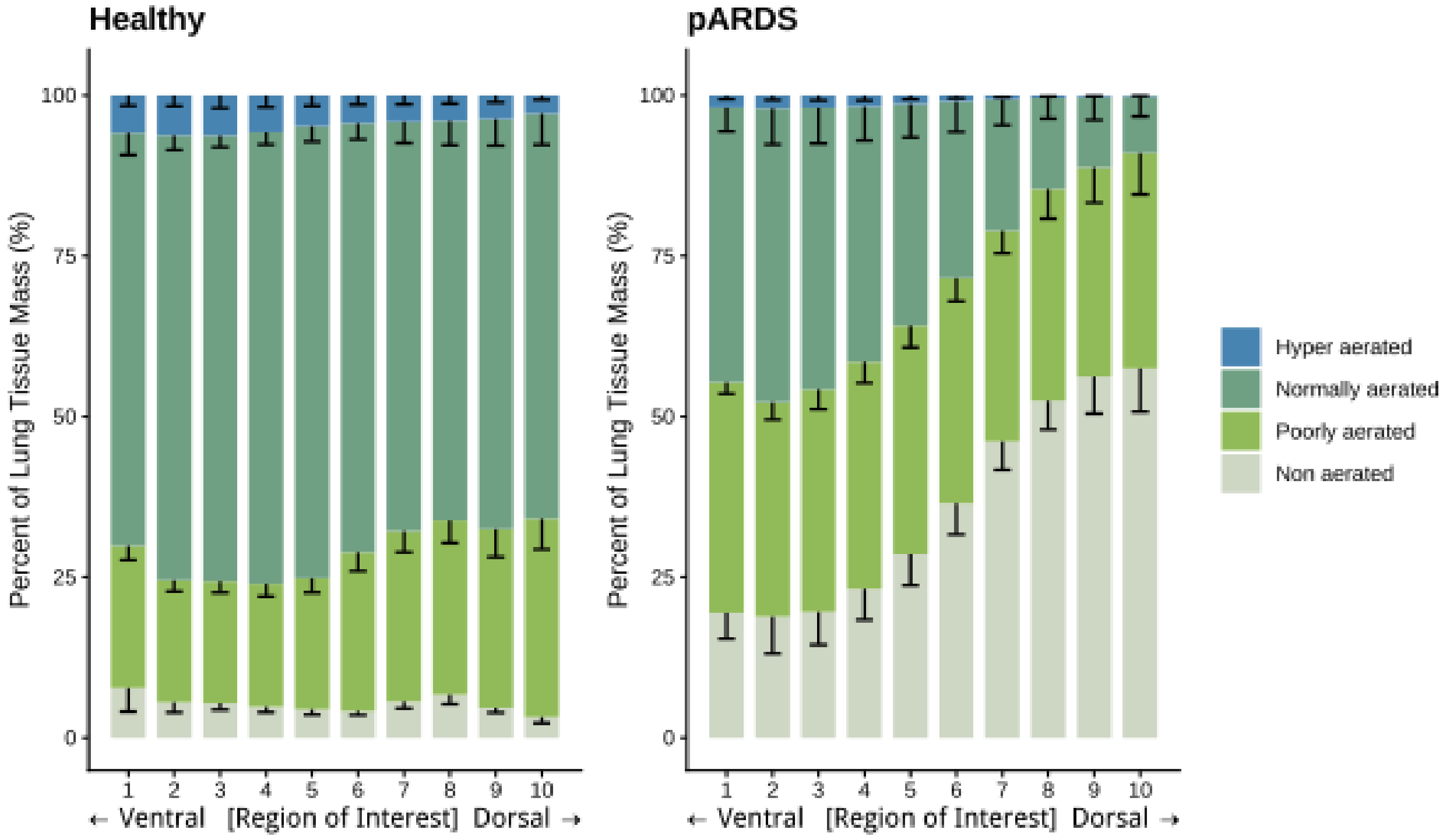

Figure S2: Percentage of total lung tissue mass classified divided in four aeration compartments for each ventral–dorsal ROI in pARDS patients and controls. Where: ROI is Region of Interest; pARDS is Pediatric Acute Respiratory Distress Syndrome.

Figure S3

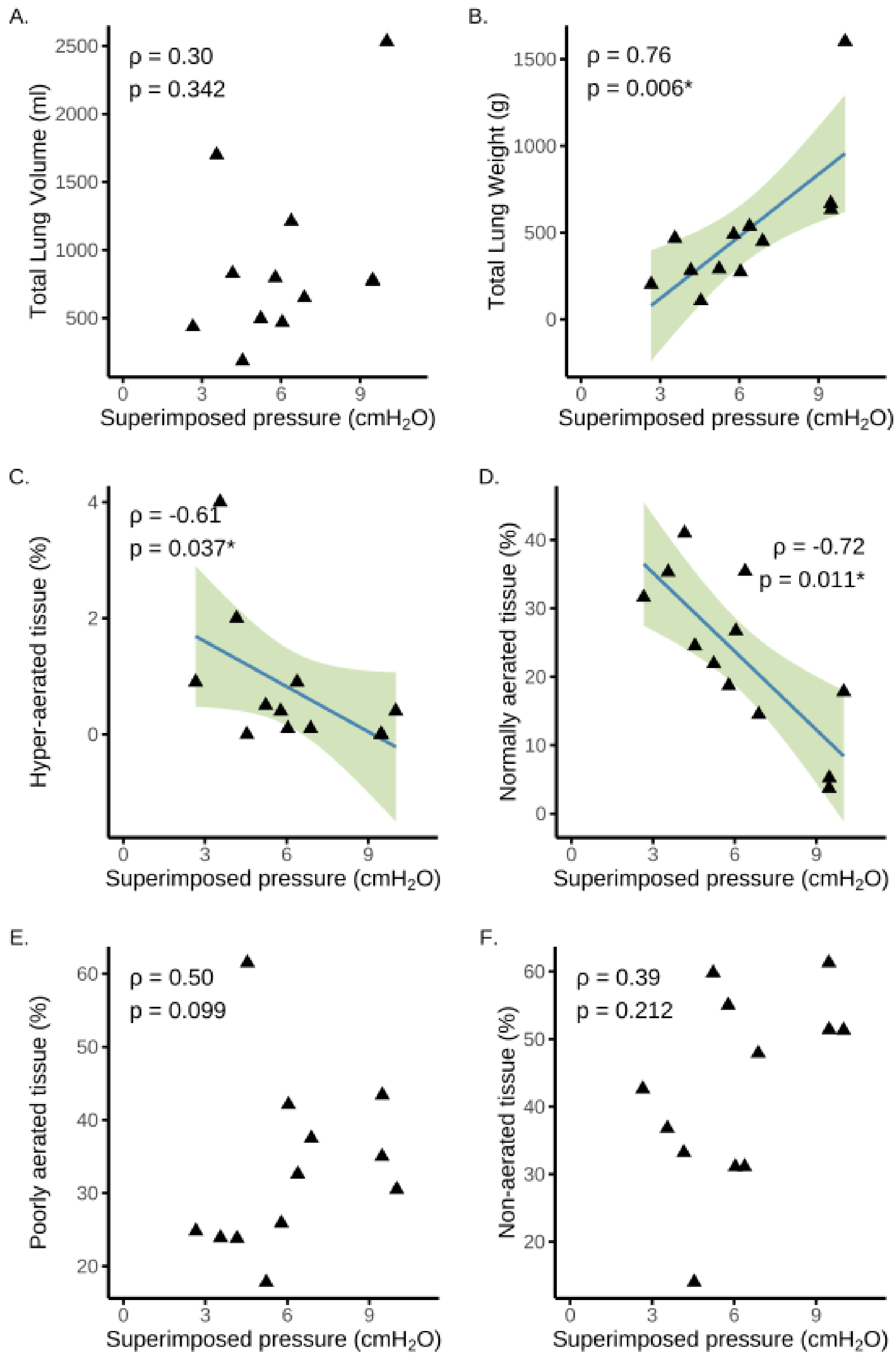

Figure S3: Scatterplots of superimposed pressure in the most dorsal ROI versus quantitative CT parameters. Lines represent linear regression with their 95% confidence bands. Where: ROI is Region of Interest; CT is Computed Tomography.

Table S1

**Variable**	**pARDS patients (N = 12)**<br><br>**Median [IQR]**
Heart rate, beats·min^{-1}	118 [98–124]
Mean arterial pressure, mmHg	67 [53–77]
Respiratory rate, breaths·min^{-1}	26 [24–29]
Inspiratory pressure, cmH_2O	22 [20–28]
Positive end-expiratory pressure (PEEP), cmH_2O	8 [6–10]
Fraction of inspired oxygen (FiO_2)	0.50 [0.40–0.70]
pH	7.38 [7.33–7.46]
PaO_2, mmHg	61 [51–69]
$PaCO_2$, mmHg	48 [41–58]
Oxygenation Index (OI)	10 [7–14]
PaO_2/FiO_2 ratio, mmHg	108 [86–150]
SpO_2/FiO_2 ratio	192 [138–238]
PaO_2/FiO_2/PEEP ratio	127 [95–273]

Table S1. Clinical and physiological characteristics of pARDS patients at the time of CT acquisition. Data are reported as median [interquartile range] and refer to the time point closest to chest CT acquisition. Where: pARDS is pediatric acute respiratory distress syndrome; CT is computed tomography; FiO_2 is fraction of inspired oxygen; PEEP is positive end-expiratory pressure; PaO_2 is partial pressure of arterial oxygen; $PaCO_2$ is partial pressure of arterial carbon dioxide; OI is oxygenation index; SpO_2 is pulse oximetry oxygen saturation; PaO_2/FiO_2 is ratio of arterial oxygen partial pressure to fraction of inspired oxygen; PaO_2/FiO_2/PEEP is PaO_2/FiO_2 ratio corrected for positive end-expiratory pressure.

Table S2

**Parameter**	**pARDS patients (N=12)**	**Controls (N=24)**	**p**
Total lung volume, ml	774 [489–924]	680 [386–2090]	0.98
Mean attenuation, HU	-415 [-542 to -352]	-700 [-816 to -611]	<0.001*
Total lung weight, g	458 [279–561]	231 [156–325]	0.045*
Total gas volume, ml	220 [182–578]	452 [258–1769]	0.045*
Gas Fraction	0.45 [0.35–0.54]	0.70 [0.61–0.82]	<0.001*
Tissue Fraction	0.59 [0.46–0.65]	0.30 [0.18–0.39]	<0.001*
Gas/Tissue ratio	0.7 [0.6–1.2]	2.3 [1.6–4.4]	<0.001*
Hyper-aerated mass, %	0.4 [0.1–0.9]	1.7 [0.4–6.0]	0.020*
Normally aerated mass, %	23.2 [17.0–32.5]	69.6 [60.6–75.0]	<0.001*
Poorly aerated mass, %	31.6 [24.6–38.7]	22.5 [17.0–34.0]	0.058
Non-aerated mass, %	45.3 [32.7–52.3]	3.5 [2.0–6.9]	<0.001*

Table S2. Results of whole-lung quantitative lung analysis. HU: Hounsfield Units. Data are reported as medians [interquartile range]. * p<0.05
